# Deep Learning Methods to Process fMRI Data and Their Application in the Diagnosis of Cognitive Impairment: A Brief Overview and Our Opinion

**DOI:** 10.3389/fninf.2018.00023

**Published:** 2018-04-26

**Authors:** Dong Wen, Zhenhao Wei, Yanhong Zhou, Guolin Li, Xu Zhang, Wei Han

**Affiliations:** ^1^Department of Software Engineering, School of Information Science and Engineering, Yanshan University, Qinhuangdao, China; ^2^The Key Laboratory of Software Engineering of Hebei Province, Yanshan University, Qinhuangdao, China; ^3^Department of Computer Science and Technology, School of Mathematics and Information Science and Technology, Hebei Normal University of Science and Technology, Qinhuangdao, China; ^4^Department of Educational Technology, College of Education, Hebei Normal University of Science and Technology, Qinhuangdao, China

**Keywords:** deep learning, fMRI, convolutional neural network, deep neural network, radial basis function network, cognitive impairment

## Introduction

In recent years, brain imaging technology has become a hot topic within the field of neuroscience. Such technology includes functional Magnetic Resonance Imaging (fMRI) (Liu et al., 2013; Wang et al., 2018), electroencephalography (EEG) (Zhang et al., 2018), Magnetoencephalography (MEG) (Alotaiby et al., 2017), and functional Near Infrared Spectroscopy (fNIRS) (Vergotte et al., 2017). In particular, because of its space resolving power, fMRI, combined with deep learning methods, has been widely used in the study of neuroscience. For example, Radial Basis Function Networks (RBFN) were used to identify Autism Spectrum Disorder (ASD) (Vigneshwaran et al., 2015), Convolutional Neural Networks (CNN) were used to diagnose amnestic Mild Cognitive Impairment (aMCI) (Meszlényi et al., 2017) and Feedforward Neural Networks (FNN) were used to classify Schizophrenia (Patel et al., 2016). When we studied this topic, we mainly searched the literature from the database of Web of Science, and reviewed references to the application of deep learning methods in the field of fMRI from 2004 to the present. This paper first summarized the analyses of the applications, advantages and disadvantages of a variety of deep learning methods in processing fMRI data, and the names, target problem, cohort size, and accuracy of these methods were presented in Table 1. Then, considering the importance of studying cognitive disorders within the field of neuroscience, this paper reviewed the trend in the development of deep learning methods to process fMRI data for the diagnosis of cognitive disorders. Lastly, recommendations for future work in developing deep learning methods to process fMRI data for the diagnosis of cognitive disorders were stated. It is expected that the analysis may provide useful suggestions to the field of the deep learning methods of fMRI data with cognitive impairment.

**Table 1:** The names, target problem, cohort size, and accuracy of deep learning methods to process fMRI data.

**Name of method**	**Target problem**	**Cohort size**	**Accuracy**	**References**
CNN	Classification of AD	AD 20, NC 20	–	Huang et al., 2017
		AD 28, NC 15	96.86%	Sarraf and Tofighi, 2016
	Classification of aMCI	aMCI 25, NC 2	71.9%	Meszlényi et al., 2017
	Classification of functional networks	HCP 68	94.61%	Zhao et al., 2017b
	Classification of mTBI	mTBI 16, HC 24	–	Zhao et al., 2017a
	Classification of the data when watching image	Sample 5275	85%	Horikawa and Kamitani, 2017a
	Classification of the data when watching video	Training set 12 Test set 9	–	Güçlü and Van Gerven, 2017
		Sample 258	72%	Wen et al., 2017
	Classification of the data when dreaming	Sample 1600	82%	Horikawa and Kamitani, 2017b
	Classification of ADHD	ADHD 274, TDC 456	69.15%	Zou et al., 2017
	Prediction of Glioma patient survival time	Glioma patient 69	89.9%	Nie et al., 2016
	Classification of the memory about word	Sample 480	73.3%	Firat et al., 2015
FNN	Identify the data in simulated war environment	Subject 5	93%	Floren et al., 2015
	Classification of the data when watching image	Subject 3	72.3%	Zafar et al., 2016
	Classification of HAND	HIV+ Subjects 5, HC 5	–	Abidin et al., 2016
	Classification of ASD	ASD 443, HC 435	70~80%	Vigneshwaran et al., 2015
		ASD 505, TC 530	70%	Heinsfeld et al., 2018
		ASD 55, TC 55	86.36%	Guo et al., 2017
	Classification of aMCI	aMCI 10, HC 8	93.75%	Jin et al., 2014
	Classification of MCI	MCI 48, NC 52	87.5%	Hu et al., 2016
	Classification of Schizophrenia	SZ 72, HC 75	85.8%	Kim et al., 2016
		SZ 72, HC 74	92%	Patel et al., 2016
	Classification of MCI	MCI 31, NC 31 MCI 12, NC 25	ADNI2 data 72.58% Own data 81.08%	Suk et al., 2016
Other methods	Classification of ADHD	Sample 263 Sample 73 Sample 113	NYU 37.41% Neuro 44.4% OHSU 80.88%	Kuang and He, 2014
	Classification of the data when execute the action	Task-fMRI data 12	94.2%	Jang et al., 2017
	Classification of the data when execute the action	Subject 20	–	Huang et al., 2016
	Classification of the data when watching video	Sample 3660	94.15%	Han et al., 2015
	Classification of the data watch different object	Sample 100	True data 100% Model data 97%	Avesani et al., 2015
	Classification of the data when read words	Sample 2160	87.5%	Kasabov et al., 2017
	Classification of ASD	ASD 539, TC 573	68.5%	Dvornek et al., 2017
	Classification of the data when touch object	Subject 4	Mean square error 0	Dixit and Mosier, 2004

*NC, normal control; TDC, typically developing children; SZ, Schizophrenia; TC, typical controls; HC, healthy controls; HIV, Human immunodeficiency virus*.

## Deep learning methods in fMRI data analysis

### CNN

In recent years, CNN has drawn much attention and has been widely used in fMRI data processing, because valid features can be automatically extracted (Sarraf and Tofighi, 2016; Horikawa and Kamitani, 2017a; Meszlényi et al., 2017). For example, CNN was used to effectively classify fMRI data (Horikawa and Kamitani, 2017a) and to classify and diagnose Alzheimer's disease (AD) (Sarraf and Tofighi, 2016) and aMCI (Meszlényi et al., 2017). The CNN method consists of the following three applications: feature extraction, construction of auto-encoder and construction of 3-Dimensional Convolutional Neural Networks (3D-CNN) method.

#### Feature extraction

To predict the lifetime of patients with brain tumors, CNN was used to extract features from fMRI data with the Support Vector Machine used for classification (Nie et al., 2016). Indeed, the study can use CNN to extract valid features, but whether the resulting features are redundant or not remains to be tested. In addition, the extracted features correspond to what features of the brain have yet to be explored.

#### Auto-encoder

Feature maps can be obtained layer by layer using an auto-encoder (Huang et al., 2017). Auto-decoders had also drawn attention when interpreting fMRI data related to vision (Wen et al., 2017). In this study, unlike in the previous studies, Deconvolutional Neural Network was constructed to achieve the operation approximate to inverse convolution. However, for this method, it is still unknown for this method to explain all the region of vision. Generally, CNN is used mainly for two-dimensional data to build an auto-encoder, but there was a 3D-Convolutional Auto Encoder built for recognition of mild Traumatic Brain Injury (mTBI) (Zhao et al., 2017a), despite more samples being needed to validate the results of the study. In order to comprehend the changes in brain state following memory words, a sparse encoder was constructed and used in conjunction with CNN for classification (Firat et al., 2015).

#### 3D-CNN

The 3D-CNN can also be used to process fMRI data. The data from Human Connectome project (HCP) was utilized to reconstruct the brain network with a higher accuracy (Zhao et al., 2017b). The methods of combining fMRI with structural magnetic resonance imaging had also been used for the classification of Attention deficit hyperactivity disorder (ADHD) (Zou et al., 2017). Although CNN can share the filter in the convolving layer and the number of parameters can be reduced in the pooling layer, the large amount of data required by the 3D network still demands extended training time.

### FNN

FNN has been applied to analyze human brain changes in a simulated war environment (Floren et al., 2015). However, the FNN method is inefficient in training based on fMRI data due to the large input eigenvector. To reduce the dimension of the data and solve this problem, the fMRI data could be passed to the input layer through the encoder (Hu et al., 2016; Kim et al., 2016). However, the method proposed by Kim et al. is not suitable for the analysis of fMRI data on patients with different diseases. Thus, its generalization on different diseases needs to be strengthened. Based on the original encoder, a Deep Auto Encoder was used to classify fMRI data with MCI (Suk et al., 2016). But this study did not consider the dynamic relationship between different regions of the brain. In another approach, four hidden layers were added in between the encoder and the decoder in the FNN method (Patel et al., 2016). Apart from adding hidden layers, multiple encoders can also be stacked. For example, when diagnosing ASD, two denoising self-encoder were constructed (Heinsfeld et al., 2018). Multiple sparse auto-encoders had also been used (Guo et al., 2017). Yet, further experiments will need to be conducted with patients of different ages to validate the clinical value of these methods.

RBFN is a FNN, which uses radial basis functions in the hidden layer to non-linearly transform the input space. RBFN had been used to study HIV Associated Neurocognitive Disorder (HAND) (Abidin et al., 2016). The RBFN could be used to extract features from fMRI data (Vigneshwaran et al., 2015). This method can be extended to studies on women of different ages. Besides the basic feature selection, a feature selection method combining region of interest and voxel was designed to determine brain states under the different stimuli (Zafar et al., 2017). With regards to cognitive diseases, RBFN had also been used to classify different regions of the brain network impaired by aMCI (Jin et al., 2014). However, the sample size in this study is limited and so the impact of larger sample sizes needs to be investigated. Meanwhile, more careful regularization methods need to be developed to address the issue of over-fitting.

### Other deep learning methods

In addition to the widely used methods described above, Restricted Boltzmann Machine (RBM) and Deep Boltzmann Machine (DBM) are also used for fMRI data analysis. The performance of the RBM had been shown to be better than independent component analysis (Huang et al., 2016), but the functional relationship between different regions of the brain was not examined. The DBM was proposed to identify areas of the brain that were activated while the subject was watching videos (Han et al., 2015). In the future, emotion-oriented data may be considered for classification. In general, the greater the number of training samples available to the deep learning method is, the better the classification performance of the method will be. Recently, three data sets (OHSU, NYU, and Neuro) used in the diagnosis of ADHD were classified with an accuracy of 80.88, 37.41, and 44.4%, respectively (Kuang and He, 2014), and the quantitative difference indicates that the generalization ability of this method is limited. When the amount of data is insufficient, the synthesis of analog data can be considered. Avesani et al. used Liquid State Machine to train and classify both simulated and real data (Avesani et al., 2015), but the results of the simulation data do not reflect the true performance of the classification method.

There are also two time-dependent deep learning methods for fMRI data analysis. The first method, Spiking Neuron Networks, converted fMRI data of the brain during reading into pulse signals (Kasabov et al., 2017). This method performed well in analyzing the temporal characteristics of the data, but failed to analyse the spatial characteristics. The second method, Long Short Term Memory (LSTM), was used for the study of ASD. In this method, the training samples were obtained from the average time series extracted from fMRI data after preprocessing (Dvornek et al., 2017). The extraction of the average time series in this study affects the accuracy of the method classification.

To reduce the number of features extracted, a deep belief network (DBN) was used to initialize the parameters and sparse pre-training was performed on weight (Jang et al., 2017). This method reduced the number of parameters and decreased the degree of over-fitting, but it takes more time for calculations.

The Hopfield Neural Network was used many years ago to classify fMRI data on subjects touching different objects (Dixit and Mosier, 2004). However, due to the small amount of data collected, the performance of the method still needs to be fully verified.

The main problem currently facing the development of deep learning methods for fMRI data analysis is that the training methods used a smaller amount of data. Therefore, the studies of small sample may lead to obvious over-fitting problems. The training methods may take more time when number of sample increase, and more training time makes it more difficult to conduct in-depth and meticulous parameter adjustment work, resulting in hindered improvement of method performance.

## Development of deep learning methods for fMRI data analysis in cognitive impairment

Cognitive disorders, which is a general category including conditions such as Alzheimer's disease, mild cognitive impairment, and subjective cognitive decline, usually encompass the disorder of memory, language, visual space, execution, calculation, and understanding, and involve multiple regions of the brain and the associated abnormalities between these brain regions. This makes the diagnosis of cognitive disorders complicated. Therefore, the diagnosis of cognitive disorders has also become a very important issue in the field of neuroscience. In the following sections, the literature analyzing deep learning methods for classification of fMRI data in cognitive impairment studies will be reviewed. Recommendations and our opinion for the development of these methods are presented.

### The measure to improve the performance of deep learning method in cognitive impairment

Firstly, for improving the model itself, as the number of fMRI data samples related to cognitive disorders is insufficient, we recommend to use a more refined regularization method, which includes the other hyperparameters of the deep learning methods. Secondly, the time cost of deep learning method training is higher, as the learning rate has an impact on the time taken for method training, the learning rate can be automatically adjusted according to the learning progress in future work. Thirdly, as the current method is aimed at studying a specific stage of the disease, a deep learning method to study all stages of the disease can be explored in the future.

From another perspective, we can apply methods for studying other diseases to cognitive impairment. In other words, deep learning methods that perform well in studying other diseases can be applied to analyzing fMRI data related to cognitive disorders. For example, the existing CNN method could be used to analyze fMRI data while the subject was watching videos (Güçlü and Van Gerven, 2017). As the method does not have to restart training, the training time is also greatly reduced.

### Exploring deep learning methods specifically for fMRI data analysis of cognitive impairment

The functioning of the brain has now been shown to involve interactions and connection among multiple functional areas, and is a phenomenon also known as brain function network dynamics (Kim et al., 2017), in which the synchronous and coupling activity will occur between or among different areas of the brain when the brain performs a task. Cognitive disorders are due to variations in the dynamic relationships between pairs of specific brain regions and the network dynamics of multiple brain regions. Therefore, as current deep learning methods disregard dynamic considerations, when designing a deep learning method for fMRI data classification of cognitive impairment, it is suggested that the training of deep learning methods could be performed after calculating the coupling or synchronizing intensity between specific brain regions.

## Conclusion

In conclusion, this study reviewed the recent literature of the deep learning used in fMRI data. Although these studies obtained promising results, more work needs to be done before conclusions can be confidently drawn in the field of deep learning used in fMRI. It must be noted that there are some limitations that occur both for deep learning and classical machine learning methods, even if in different proportions. For example, a small sample size is a source of overfitting in both cases, even if the number of samples required for training a classical machine learning method is extremely lower than the one needed for deep learning techniques (Salvatore et al., 2016). In addition, brain dynamics have not been taken seriously in the two ways. Compared with the classical machine learning methods, deep learning methods may have poorer performance under the condition of small sample size and training time cost (Nie et al., 2016), but we can make full use of the automatically-extracted features to improve accuracy of deep learning methods. In the future, we suggest collecting more subjects of cognitive impairment to test and revise the existing methods, and considering adequately the dynamic relationships among different brain regions.

## Author contributions

DW: designed the study and wrote this paper; ZW and YZ: analyzed literatures and wrote this paper; GL and XZ: revised this paper; WH: designed the study.

### Conflict of interest statement

The authors declare that the research was conducted in the absence of any commercial or financial relationships that could be construed as a potential conflict of interest.
